# Nucleic acids and proteins carried by exosomes of different origins as potential biomarkers for gynecologic cancers

**DOI:** 10.1016/j.omto.2021.12.005

**Published:** 2021-12-09

**Authors:** Miaomiao Ye, Jing Wang, Shuya Pan, Lihong Zheng, Zhi-Wei Wang, Xueqiong Zhu

**Affiliations:** 1 Department of Obstetrics and Gynecology, The Second Affiliated Hospital of Wenzhou Medical University, No. 109 Xueyuan Xi Road, Wenzhou, Zhejiang 325027, China

**Keywords:** exosomes, nucleic acids, proteins, ovarian cancer, cervical cancer, endometrial cancer

## Abstract

Exosomes are extracellular vesicles with a diameter of 30–150 nm that function in mediating intercellular communication and intercellular material exchange. The liposomal membrane of exosomes protects the cargo carried by exosomes from degradation and assists in transporting cargo to recipient cells to regulate a variety of physiological and pathological processes. The incidence of gynecologic cancers is increasing annually, which is extremely harmful to the lives and health of women because such cancers are challenging to detect at the early stage. Recently, exosomes have emerged as novel biomarkers for diagnosing and predicting the development of gynecologic cancers. In particular, non-coding RNAs (microRNAs [miRNAs], long non-coding RNAs [lncRNAs], and circular RNAs [circRNAs]) carried by exosomes have been extensively investigated in gynecologic cancers. Therefore, the purpose of this review is to focus on the potential roles of exosomes of different origins in ovarian cancer, cervical cancer, and endometrial cancer, which will help to determine the molecular mechanism of carcinogenesis.

## Graphical abstract

**Figure:**
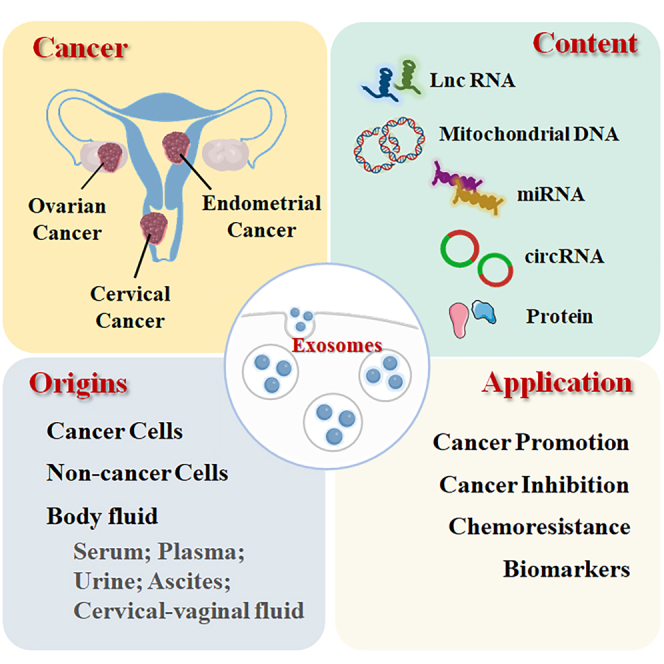

## Teaser

Exosomes can mediate intercellular communication and material exchange. This review focuses on the potential roles of exosomes of different origins in ovarian cancer, cervical cancer, and endometrial cancer, which will determine the molecular mechanisms of carcinogenesis and discover the potential biomarkers for gynecologic cancers.

## Introduction

Gynecologic cancers, such as ovarian, cervical, and endometrial cancers, significantly contribute to the global cancer burden.^1^ For instance, ovarian cancer is one of the leading causes of death in women. At all stages, the 5-year overall survival rate of ovarian cancer patients is approximately 47%, and more than 70% of ovarian cancer patients are diagnosed at advanced stages, and the 5-year overall survival rate is even lower.^2^^,^^3^ Cervical cancer is the fourth most common female cancer worldwide and remains a major health problem, especially for women in developing countries.^4^ Endometrial cancer is commonly referred to as a type of uterine cancer that develops from the inner lining of the uterus, mainly occurring in postmenopausal women.^5^ Advanced and recurrent gynecologic cancers are associated with poor prognosis and lack of effective clinical treatments.^5^ Hence, early detection of cancers can contribute to improving the survival rate of gynecologic cancer patients, and the use of novel available technologies to identify promising biomarkers for these cancer patients must be considered.

Extracellular vesicles (EVs) are small particles composed of lipid bilayer membranes that are released by diverse cells in both physiological and pathological states.^6^ EVs are primarily classified into three categories, namely, apoptotic bodies (1,000–5,000 nm in diameter), microvesicles (200–1,000 nm in diameter), and exosomes (30–150 nm in diameter), according to their biogenesis.^7^^,^^8^ Apoptotic bodies are fragments released from apoptotic cells containing fragmented DNA, and cell organelles from their host cells can be found.^9^ Microvesicles bud directly from the plasma membrane of various cells.^10^ Exosomes are secreted from multivesicular bodies (MVBs) after fusion with the plasma membrane.^11^^,^^12^ Exosomes have a double-membrane structure with an abundance of cargo contents, such as proteins, messenger RNAs (mRNAs), long non-coding RNAs (lncRNAs), microRNAs (miRNAs), lipids, and viral particles, and exhibit a dish-like or cup-shaped morphology under electron microscopy.^13^^,^^14^ Exosomes can be isolated from body fluids, such as blood plasma or serum, ascites, saliva, urine, ejaculate, and human breast milk, and are released by numerous cell types, including cancer cells, mesenchymal stem cells, and immune cells.^15^, ^16^, ^17^ At present, ultracentrifugation (UC) is a commonly applied technique to isolate exosomes and can be conducted in one of the two ways: differential based or density gradient based.^18^^,^^19^

Exosomes serve as an important tool for intercellular communication and intercellular material exchange and can transfer informative substances to neighboring cells or even distant cells.^20^ The exosome-based cargo delivery system functions in intercellular communication through endocytosis and receptor-ligand interactions, and subsequently participates in regulating a variety of physiological and pathological processes.^21^^,^^22^ In particular, compared with healthy cells, the cancer cells release more exosomes, the molecular compositions, cargo, and properties of which differ from those of exosomes secreted by healthy cells.^23^^,^^24^ To date, the essential role of exosomes in carcinogenesis and tumor progression has been studied extensively.^25^^,^^26^ Remarkably, cancer-cell-derived exosomes can provide a suitable microenvironment for cancer development via regulation of cell proliferation,^27^^,^^28^ angiogenesis and metastasis,^29^ immune modulation,^30^ and drug resistance.^31^ Moreover, exosomes released by primary ovarian cancer serve as coordinators for the establishment of a premetastatic niche by inducing immunosuppression, facilitating angiogenesis, and remodeling stromal cells within the premetastatic niche of ovarian cancer.^32^

**Figure 1:**
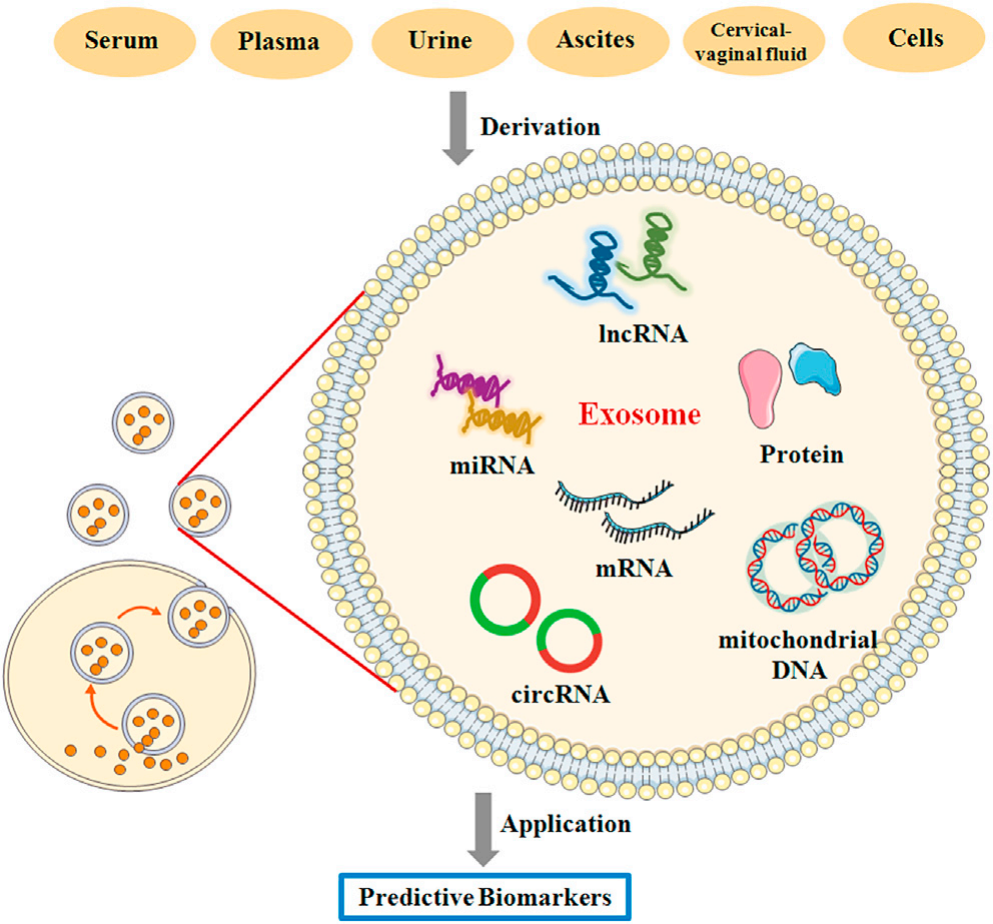
Exosome-carried nucleic acids and proteins as potential predictive biomarkers in gynecologic cancers

The nucleic acids and proteins carried by exosomes are packaged in the liposomal membrane, which protects the cargo from degradation, and potentially provides an essential source of clinical information for research.^33^^,^^34^ Non-coding RNAs (ncRNAs) are critical components carried by exosomes, and positively function in diverse biological processes.^35^ ncRNAs consist of various RNA transcripts, encompassing miRNAs, lncRNAs, and circular RNAs (circRNAs).^36^ Notably, more than 50% of miRNA genes are localized in tumor-related genomic loci or fragile regions, exerting a regulatory effect on carcinogenesis.^37^ lncRNAs are characterized by a low expression level, poor conservation between species, and a high coefficient of variation.^38^ Additionally, lncRNAs are involved in the regulation of tumor angiogenesis, cancer cell stemness, and acquired resistance to chemotherapy by serving as a competitive endogenous RNAs (ceRNAs) and binding directly to related proteins or mRNAs.^35^^,^^38^ CircRNAs are novel ncRNA molecules with a closed-loop structure owing to the covalent linking of 5′ and 3′ termini, and the knowledge of their canonical role remains at a superficial level.^39^ Acting as an miRNA sponge is the potential mechanism by which circRNAs are currently believed to promote tumor progression.^40^ Therefore, the roles of exosome-carried nucleic acids and proteins in ovarian cancer, cervical cancer, and endometrial cancer have been summarized in this review (Figure 1). The effects of exosomes on cancer biology will contribute to determining the molecular mechanism of carcinogenesis and discovering the potential biomarkers for gynecologic cancers.^32^^,^^41^

## The role of exosomes in ovarian cancer

Ovarian cancer is considered the most aggressive gynecologic malignancy and exhibits high genetic complexity and heterogenicity, with epithelial ovarian cancer (EOC) accounting for 85% of all cases.^42^^,^^43^ Although serum cancer antigen 125 (CA125) detection and ultrasonography are routine methods for the clinical diagnosis of ovarian cancer, they are not suitable for early detection because of low specificity and sensitivity.^44^^,^^45^ In recent years, studies have revealed that exosome-mediated transport of nucleic acids (miRNAs, mRNA, mitochondrial DNA, lncRNAs, and circRNAs) is involved in ovarian carcinogenesis, especially nucleic acids isolated from the plasma or serum of patients.^46^^,^^47^

## The role of exosome-carried nucleic acids in ovarian cancer

### Role of ovarian cancer-cell-derived exosomes in ovarian cancer

Miharu et al.^48^ conducted a study on highly (SKOV-3) and weakly (OVCAR-3) invasive ovarian cancer cell lines, and determined that SKOV-3 cells released 2.7-fold more exosomes than OVCAR-3 cells. Moreover, SKOV-3-cell-derived exosomes had a higher expression of let-7 family transcripts than that in the OVCAR-3-cell-derived exosomes, while the expression of miR-200 family transcripts was detected only in OVCAR-3-cell-derived exosomes.^48^

Exosomal miR-99a-5p from EOC cells improved the invasive ability of EOC cells through upregulation of fibronectin and vitronectin in human peritoneal mesothelial cells.^49^ Ovarian cancer-cell-secreted exosomal miR-205 accelerated metastasis by promoting angiogenesis by regulating the phosphatase and tensin homolog (PTEN)/Akt pathway.^50^ Additionally, SKOV3-cell-derived exosomal miR-205 governed proliferation, migration, invasion, and apoptosis via inhibition of vascular endothelial growth factor A (VEGFA) in ovarian cancer cells.^51^ Similarly, exosomes originating from SKOV-3 cells strengthened the metastatic ability of ovarian cancer compared with the exosomes from OVCAR-3 cells, which was confirmed in an ovarian cancer cell xenograft mouse model.^52^ Furthermore, exosomal circPUM1 derived from CAOV3 ovarian cancer cells promoted tumor metastasis of peritoneal mesothelial cells via upregulation of nuclear factor (NF)-κB and matrix metallopeptidase 2 (MMP2) expression.^53^ CircWHSC1 promoted ovarian oncogenesis, and its exosome forms isolated from CAOV3 cells promoted metastasis by acting on peritoneal mesothelium by upregulating mucin 1 (MUC1).^54^ Taken together, the nucleic acids carried by exosomes derived from ovarian cancer cells potentially influence the invasive and metastatic abilities of ovarian cancer.

Exosomes derived from ovarian cancer cells promoted angiogenesis in serous ovarian cancers, especially in the high-grade ovarian cancers.^55^ Additionally, exosomes originating from SKOV3-DDP (cisplatin) cells were abundant in miR-130a, which facilitated angiogenesis.^56^ Higher miR-940 expression in exosomes isolated from EOC cells was induced by hypoxia, and exosomal miR-940 was also abundant in the ascites of EOC patients.^57^ Moreover, tumor-derived exosomal miR-940 exhibited a vital role in promoting the polarization of tumor-associated macrophages in EOC progression.^57^ Exosomal miR-1246 was highly expressed in ovarian cancer-cell-derived exosomes, which exerted oncogenic properties in the tumor microenvironment by transferring the miR-1246 to the M2-type macrophages and subsequently had the potential to downregulate the expression of caveolin 1 (Cav1).^58^ Exosomes derived from human ovarian surface epithelial cells transferred miR-124 to cancer-associated fibroblasts (CAFs), which suppressed the transition from normal fibroblasts to cancer-associated fibroblasts by repressing sphingosine kinase 1 (SPHK1) expression in ovarian cancer.^59^ The above studies suggested that nucleic acids carried by exosomes derived from ovarian cancer cells function in the regulation of the tumor microenvironment.

### Role of non-tumor-cell-derived exosomes in ovarian cancer

Exosomes purified from primary-cultured omental fibroblasts of ovarian cancer patients were loaded with miR-199a-3p (miR-199a-3p-Exo), and miR-199a-3p-Exo downregulated the expression of c-Met, which subsequently suppressed the proliferation and invasion of ovarian cancer cells.^60^ In addition, miR-199a-3p-Exo decreased the peritoneal dissemination in an ovarian cancer mouse model, and reduced the expression of c-Met, extracellular signal-regulated kinase (ERK) phosphorylation, and MMP2 in cancers.^60^ Exosomal miR-7 purified from the tumor necrosis factor (TNF)-like weak inducer of apoptosis (TWEAK)-stimulated macrophages (TMs) suppressed the metastatic ability of EOC cells and inhibited the epidermal growth factor receptor (EGFR)/AKT/ERK1/2 signaling pathway.^61^ Nevertheless, exosomes isolated from some cell types promoted the progression of ovarian cancer. Plasma cells conferred the mesenchymal identity of ovarian cancers by enhancing the transfer of exosome-derived miR-330-3p.^62^ Exosomal miR-98-5p from CAFs facilitated the proliferation and cell cycle entry and inhibited the apoptotic ability of ovarian cancer cells.^63^ In summary, the different characteristics of exosomes in ovarian cancer development are predominantly determined by the cell types from which they originate.

### Role of exosomes derived from body fluids in ovarian cancer

Xu et al.^64^ found that the expression of miR-101 and miR-30a was downregulated, while the expression of miR-21 and miR-210 was upregulated, in ovarian cancer tissues versus the noncancerous tissues. However, only miR-101 expression was significantly downregulated in the serum exosomes from ovarian cancer patients, suggesting that miR-101 potentially serves as a biomarker for ovarian cancer.^64^ The expression of miR-146b-5p in exosomes from the serum of EOC patients was higher than that in exosomes from healthy individuals.^65^ Zhang et al.^66^ documented that plasma exosomal miR-106a-5p was upregulated, while plasma exosomal miR-122-5p and miR-185-5p were downregulated, in ovarian cancer patients compared with healthy individuals. One study using microarrays found that six circRNAs were increased and 37 circRNAs were decreased in serum exosomes from ovarian cancer patients.^67^ Among the six circRNAs, higher circ-0001068 expression in the serum exosomes was further confirmed in a large cohort.^67^ Circ-0001068 induced programmed death 1 (PD1) expression by targeting miR-28-5p after it was delivered into T cells by exosomes.^67^ Different expression levels of nucleic acids in serum or plasma exosomes were found not only between ovarian cancer patients and healthy individuals but also among ovarian cancer patients with different grades. A study revealed that serum exosomal miR-34a expression was elevated in early-stage ovarian cancer cases.^68^ Consistently, exosomal miR-34a in serum was downregulated in ovarian cancer patients with lymph node metastasis and recurrence.^68^ MiR-214-3p, which is an epigenetic regulator with an oncogenic role in EOC,^69^ was highly expressed in serum exosomes from patients with highly malignant EOC and platinum-resistant high-grade serous ovarian cancer (HGSOC).^70^ This study also demonstrated that the miR-214-3p and its target gene, LIM homeobox domain 6 (LHX6), in serum exosomes potentially acted as biomarkers for predicting EOC progression.^70^ Moreover, Keseru et al.^71^ detected the exosome-encapsulated mitochondrial DNA (mtDNA) copy number in the cell-free plasma of serous epithelial ovarian cancer patients, and indicated that the exosomal mtDNA copy number was increased in late-stage ( International Federation of Gynecology and Obstetrics [FIGO] stages III and IV) patients compared with the healthy individuals.

Elevated expression of miR-1307 and miR-375 was found in serum exosomes from ovarian cancer patients in contrast to that in patients with benign ovarian tumors and healthy individuals, which may improve the diagnostic efficiency of CA125 for ovarian cancer.^72^ One study detected miR-93, miR-145, and miR-200c upregulation in exosomes derived from the serum of ovarian cancer patients, and the triple combination of serum exosomal miR-145 and miR-200c and serum CA125 was the most effective biomarker, with a sensitivity of 100% for the differential diagnosis of ovarian masses (benign ovarian cysts/borderline ovarian neoplasms versus ovarian cancers), indicating the strong diagnostic potential of miRNAs carried by exosomes.^73^ Lower expression of serum exosomal miR-484 was found in ovarian cancer patients than in healthy individuals, and detection of serum exosomal miR-484 with CA125 exhibited a good diagnostic performance in discriminating ovarian cancer patients and healthy individuals, with an area under the receiver operating characteristic (ROC) curve (AUC) of 0.912.^74^ Taken together, the combination of serum CA125 and nucleic acids in blood exosomes of ovarian cancer patients potentially enhances the diagnostic efficiency in ovarian cancer.

Exosomal miR-21, miR-100, miR-200b, and miR-320 were highly expressed, while exosomal miR-16, miR-93, miR-126, and miR-223 were weakly expressed, in the plasma of EOC patients compared with healthy individuals.^75^ In particular, the elevated expression of exosomal miR-200b was associated with an increasing value of CA125 and a poor overall survival for EOC patients.^75^ Additionally, ovarian cancer patients simultaneously with a low serum exosomal miR-484 and a high serum CA125 expressions showed a trend toward worse clinical outcomes.^74^ The upregulation of serum exosomal lncRNA metastasis-associated lung adenocarcinoma transcript 1 (MALAT1) in EOC patients was associated with an advanced and metastatic phenotype of EOC, which potentially predicted poor overall survival for EOC patients.^76^ Therefore, the nucleic acids carried by blood exosomes of ovarian cancer patients may act as biomarkers for predicting their clinical outcomes.

Apart from plasma or serum, exosomes in other body fluids have attracted the attention of many researchers. miRNA microarray data revealed that miR-30a-5p expression was upregulated in the urine of serous ovarian adenocarcinoma patients compared with healthy controls and demonstrated that the urinary miR-30a-5p originated from the exosomes excreted by ovarian cancer cells.^77^ Cappellesso et al.^78^ confirmed that the mRNA expression of programmed cell death 4 (PDCD4) was upregulated, while miR-21 expression was downregulated, in the cells and exosomes from peritoneal effusions of serous ovarian carcinoma in contrast to nonneoplastic peritoneal effusions. This study also indicated that miR-21 transfer by exosomes could facilitate carcinogenic transformation in target cells far from the primary tumor without direct colonization by cancer cells and can be regarded as a diagnostic tool for ovarian serous carcinoma.^78^ Exosomes from ascites of ovarian cancer patients transferred miR-6780b-5p to ovarian cancer cells, which facilitated epithelial-mesenchymal transition (EMT) and subsequently promoted ovarian cancer metastasis.^79^

## The role of exosome-carried proteins in ovarian cancer

Exosomes can package and release the unique proteins associated with a disease; therefore, the identification of characteristic proteins in the exosomes originating from ovarian cancer patients holds promise for the diagnosis of ovarian cancer.^80^ High cluster of differentiation (CD) 24 expression in plasma exosome was reported in serous ovarian cancer patients.^81^ CD24 and epithelial cell adhesion molecule (EpCAM) proteins were identified in exosomes purified from ascites fluid of ovarian cancer patients.^82^ Microfluidic technology can realize the rapid separation of blood exosomes^83^, ^84^, ^85^; in particular, microfluidic technology can validate significant biomarkers, such as folate receptor alpha (FRα) in plasma exosomes from ovarian cancer patients^86^ and hepatocyte growth factor (HGF), STAT3, and interleukin 6 (IL-6) in serum exosome samples from early-stage HGSOC patients.^87^ Zhang et al.^88^ identified 294 proteins in plasma exosomes from EOC patients and healthy individuals, and demonstrated that the lipopolysaccharide-binding protein (LBP), gelsolin, fibrinogen gamma chain, and fibrinogen alpha chain proteins in plasma exosomes from EOC patients could be used as biomarkers for the diagnosis of ovarian cancer. Furthermore, the plasma of ovarian cancer patients carried higher levels of exosomal proteins, including melanoma-associated antigen 3/6 (MAGE 3/6) and transforming growth factor β 1 (TGF-β1), than that of ovarian benign tumor patients and healthy individuals, which provided a novel strategy to diagnose ovarian cancer patients.^89^

Studies have demonstrated that exosome-mediated transfer of key proteins plays an essential role in ovarian carcinogenesis.^90^^,^^91^ For instance, the migration and invasion of low-metastatic ovarian cancer cells were enhanced due to exosome-involved transfer of CD44 from high-metastatic ovarian cancer cells.^90^ Exosomes derived from ovarian cancer cells transported CD44 to human peritoneal mesothelial cells, thus increasing the invasive ability of ovarian cancer.^92^ Serum exosomal antisense hypoxia inducible factor (aHIF) was highly expressed in EOC patients and was associated with poor overall survival, indicating that exosomal aHIF might be a prognostic predictor for EOC.^93^

Recently, researchers applied proteomics techniques to identify the promising biomarkers in ovarian cancer. Bebelman et al.^94^ observed high levels of the proteins glucose-6-phosphate dehydrogenase, transketolase, and transaldolase, which are key regulatory enzymes in the pentose phosphate pathway, in exosomes originating from two late-stage ovarian cancer cell lines, OVCA429 and HO8910PM. Liang et al.^95^ conducted a nano liquid chromatography-tandem mass spectrometry (LC-MS/MS) workflow on the exosomes from OVCAR-3 and IGROV1 ovarian cancer cell lines, and identified 2,230 exosomal proteins in these two ovarian cancer cell lines. Moreover, another study used an ExoProfile chip to analyze the circulating exosomes from the plasma of ovarian cancer patients, and revealed that the combinations of multiple markers, including EGFR, human EGFR 2 (HER2), CA125, folate receptor α (FRα), CD24, EpCAM, CD9, and CD63, in circulating exosomes potentially improved the efficacy for differentiating early- and late-stage ovarian cancer, yielding the best diagnostic AUC value.^96^ In addition, another study revealed that 1,433 proteins and 1,227 lipid species were identified in exosomes from the SKOV3 ovarian cancer cell line and HOSEPiC ovarian surface epithelial cell line, and demonstrated that the lipids cholesterol ester (ChE) and zymosterol (ZyE) species and collagen type V alpha 2 chain (COL5A2) and lipoprotein lipase (LPL) proteins were more abundant in exosomes derived from SKOV3 ovarian cancer cells than those derived from HOSEPiC ovarian surface epithelial cells.^97^

## The role of exosomes in regulating chemotherapy of ovarian cancer

The exosomal tumor suppressor miR-6126 was detected at high levels in both chemosensitive and chemoresistant ovarian cancer cells.^98^ Higher expression of lncRNA urothelial carcinoma-associated 1 (UCA1) was detected in the serum exosomes of cisplatin-resistant ovarian cancer patients than their cisplatin-sensitive counterparts.^99^ One study revealed a lower expression of the circRNA Cdr1as in the serum exosomes of cisplatin-resistant ovarian cancer patients than in cisplatin-sensitive patients.^100^ The above studies indicated that the expression levels of nucleic acids in exosomes differed between chemosensitive and chemoresistant ovarian cancers.

Serum exosomal circular forkhead box protein P1 (circFoxp1) was upregulated in EOC patients, especially in DDP-resistant EOC patients, which conferred cisplatin resistance in EOC cells.^101^ Exosomal DNA methyltransferase 1 (DNMT1) transcripts were highly expressed in exosomes derived from ovarian cancer cells and enhanced the cisplatin resistance of ovarian cancer cells.^102^ In addition, miR-21-3p, miR-21-5p, and miR-891-5p were abundant in exosomes derived from ovarian cancer cells, contributing to carboplatin resistance of ovarian cancer cells.^103^ In particular, ovarian cancer patients at risk of relapse showed higher miR-891-5p expression of exosomes.^103^ Macrophage-derived exosomes transferred miR-223 to EOC cells to trigger chemoresistance via regulation of the PTEN/PI3K/Akt pathway, and the circulating exosomal miR-223 expression was linked to EOC recurrence.^104^ Exosomal miR-98-5p from CAFs enhanced cisplatin (DDP) resistance via downregulation of cyclin-dependent kinase inhibitor 1A (CDKN1A) in subcutaneous-ovarian-cancer-bearing nude mice.^63^ Exosomal plasma gelsolin enhanced the survival of ovarian cancer cells via both autocrine and paracrine mechanisms to enhance chemoresistance.^91^ The enhanced expression of exosome-carried plasma gelsolin from ovarian cancer cells weakened the immunosurveillance and promoted the synthesis of glutathione, which subsequently caused chemoresistance in ovarian cancer.^105^ Moreover, miR-21 transferred by exosomes isolated from neighboring stromal cells conferred paclitaxel resistance to ovarian cancer cells via regulation of apoptotic peptidase activating factor 1 (APAF1).^106^

Exosomal miR-146a derived from human umbilical cord mesenchymal stem cells (hUCMSCs) inhibited the growth and ameliorated the chemoresistance of ovarian cancer cells.^107^ Moreover, silencing of miR-146a in hUCMSC-derived exosomes promoted the growth and chemoresistance of ovarian cancer cells through the phosphatidylinositol 3-kinase (PI3K)/Akt signaling pathway via laminin γ2.^107^ Exosomes released by miR-30a-5p mimic-transfected SKOV3/DDP cells inhibited the proliferative ability and enhanced the apoptotic ability of SKOV3 cells, and improved the chemosensitivity of SKOV3 cells to DDP by downregulating the expression of SRY-box 9 (SOX9).^108^ Taken together, the different expression levels of nucleic acids and proteins in exosomes potentially contributed to the chemoresistance or chemosensitivity of ovarian cancer, and these biomarkers may become targets for clinical therapy.

Cisplatin-loaded exosomes from umbilical cord blood-originated M1 macrophages exerted a superior inhibitory effect on the growth of the A2780 EOC cell line and cisplatin-resistant A2780/DDP cell line compared with free cisplatin, and ameliorated the cisplatin resistance of ovarian cancer cells.^109^ Exosomes derived from SKOV3 cells were loaded with triptolide, and the triptolide-loaded exosomes exerted a stronger inhibitory effect on proliferation and growth and exhibited weaker cytotoxic and apoptotic effects on SKOV3 cells than did free triptolide.^110^ The exosome-based drug delivery system showed better therapeutic effects than a single drug in ovarian cancer.

## The role of exosomes in cervical cancer

Cervical cancer can be broadly classified into two categories: cervical squamous cell carcinoma (accounting for 80% of cases) and cervical adenocarcinoma (accounting for 9% of cases).^111^ Persistent high-risk human papillomavirus (HPV) infection is considered a leading cause of cervical cancer, and the HPV oncogenes E6 and E7 play an essential role in this process.^112^^,^^113^ However, a few patients with cervical cancer do not show HPV infection; thus, preventing high-grade cervical intraepithelial neoplasia (CIN) from developing into invasive cervical cancer through early screening is crucial.^114^

### Role of cervical-cancer-cell-derived exosomes in cervical cancer

One study reported that miR-221-3p in exosomes originating from cervical cancer cells promoted the proliferation, invasion, migration, and angiogenesis of microvascular endothelial cells in cervical cancer by reducing the expression of mitogen-activated protein kinase 10 (MAPK10).^115^ Cervical squamous cell carcinoma-cell-derived exosomes delivered miR-221-3p from cancer cells to the human umbilical vein endothelial cells (HUVECs), and the exosomal miR-221-3p promoted angiogenesis via downregulation of Thrombospondin-2 in cervical squamous cell carcinoma.^116^ Cervical cancer cells exposed to TGF-β1 tended to secrete more miR-663b-containing exosomes, and exosomal miR-663b could be endocytosed by the adjacent or distant cervical cancer cells.^117^ Exosomal miR-663b reduced the expression of mannoside acetyl-glucosaminyltransferase 3 (MGAT3), which subsequently facilitated the EMT and enhanced the local and distant metastasis of cervical cancers.^117^ One study illustrated that knockdown of HPV E6/E7 expression in cervical cancer cells (HPV18-positive HeLa cells) contributed to elevated release of HeLa--cell-derived exosomes.^118^ Exosomal let-7d-5p, miR-20a-5p, miR-378a-3p, miR-423-3p, miR-7-5p, and miR-92a-3p were downregulated, while exosomal miR-21-5p was upregulated in exosomes isolated from HeLa cells with the silencing of E6/E7 silencing.^119^ Cervical cancer-cell-secreted exosomes carrying lncRNA HNF1A-AS1 facilitated the proliferation and DDP resistance and weakened the apoptosis of cervical cancer cells via the upregulation of Tuftelin1 and downregulation of miR-34b.^120^ Cervical cancer cells transfer the hedgehog pathway (Hh)-related proteins (Patched1, Smoothened, Sonic hedgehog, Indian hedgehog) in their exosomes.^121^ Exosomal Wnt2B protein from cervical cancer cells promoted the activation of fibroblasts, thereby facilitating the progression of cervical cancer.^122^ Taken together, nucleic acids and proteins carried by exosomes released by cervical cancer cells potentially exert a pro-oncogenic effect on cervical cancer.

### Role of non-tumor-cell-derived exosomes in cervical cancer

MiR-155-5p in exosomes isolated from HIV-infected T cells facilitated the proliferative, migrative, and invasive abilities of cervical cancer cells, which may be mediated through the AT-rich interaction domain 2 (ARID2)-ERCC5-NF-κB pathway.^123^ Furthermore, miR-22-enriched exosomes originating from the pre-miR-22-transfected HEK293 cells improved the sensitivity of cervical cancers to radiotherapy.^124^ More research must be carried out to study the effects of non-tumor-cell-derived exosomes in cervical cancer.

### Role of exosomes derived from body fluids in cervical cancer

One study indicated that the expression of miR-146a-5p, miR-151a-3p, and miR-2110 was elevated in plasma exosomes derived from cervical cancer patients in contrast to those from healthy individuals.^125^ Plasma exosomal miR-125a-5p showed higher expression in cervical cancer patients than in the healthy individuals and potentially acted as a marker for the differential diagnosis between cervical cancer patients and healthy individuals.^126^ Moreover, Zheng et al.^127^ demonstrated that eight differentially expressed plasma exosomal miRNAs (let-7a-3p, let-7d-3p, miR-30d-5p, miR-144-5p, miR-182-5p, miR-183-5p, miR-215-5p, and miR-4443) could discriminate CIN II+ patients (including advanced CIN II patients) from CIN I patients (including CIN I patients and healthy individuals). However, only let-7d-3p and miR-30d-5p showed significant differences between cervical cancer tissues and adjacent normal tissues,^127^ indicating that exosomal miRNAs might be selectively secreted by tumor cells. Higher expression of serum exosomal lncRNA DLX6-AS1 was detected in cervical cancer patients than in the CIN patients and healthy individuals, which was positively correlated with lymph node metastasis, differentiation, and FIGO stage, and predicted relapse and a worse clinical outcome for cervical cancer patients.^128^ Altogether, the exosomes isolated from the plasma or serum of cervical cancer patients are useful in cervical cancer screening and diagnosis.

One study documented that the expression of PI3k/Akt/mammalian target of rapamycin (mTOR) in cervical cancer tissues and in the exosomes derived from vaginal secretions was higher than that in the adjacent normal cervical tissues, but the expression levels did not significantly differ between cervical cancer tissues and the exosomes derived from vaginal secretions.^129^ Additionally, exosomal miR-146a and miR-21 separated from cervicovaginal lavage fluid were increased in cervical cancer patients compared with individuals with no (pre)cervical disease (with or without HPV infection).^130^ Compared with that in cancer-free volunteers (with or without HPV infection), the expression of exosomal lncRNAs hox transcript antisense intergenic RNA (HOTAIR) and metastasis-associated lung carcinoma transcript 1 (MALAT1) was upregulated, whereas the expression of exosomal lncRNA maternally expressed gene 3 (MEG3) was downregulated in the cervicovaginal lavage fluid of cervical cancer patients.^131^ Moreover, among the cancer-free volunteers, elevated expression of exosomal lncRNAs HOTAIR and MALAT1 and decreased expression of exosomal lncRNA MEG3 were detected in the cervicovaginal lavage fluid from HPV-positive individuals compared with those in HPV-negative individuals, indicating that lncRNAs HOTAIR, MALAT1, and MEG3 may have an important role in the development of cervical cancer.^131^ Additionally, 45 miRNAs were upregulated and 55 miRNAs were downregulated in exosomes purified from the cervical-vaginal fluid of HPV16-infected patients versus those in the HPV16-free individuals.^132^ The detection and analysis of cervicovaginal lavage samples and vaginal secretions of cervical cancer patients, which are rich in exosomes, provide a new idea for noninvasive cervical cancer screening.^130^

## The role of exosomes in endometrial cancer

Endometrial cancer predominantly occurs in postmenopausal women and can be grouped into two clusters: estrogen dependent (type I) and estrogen independent (type II).^133^^,^^134^ miRNAs are aberrantly expressed in endometrial cancer, and dysregulated miRNAs potentially function as pro-oncogenesis factors or tumor suppressors in endometrial cancer progression.^135^^,^^136^

### Role of exosomes derived from cells in endometrial cancer

Endometrial cancer cells can deliver small regulatory RNAs to endometrial fibroblasts via exosomes.^137^ Exosomal miR-133a was found in the exosomes derived from endometrial cancer cells and could be transferred to the normal endometrial cells.^138^ Exosomal lncRNA deleted in lymphocytic leukemia1 (DLEU1) derived from endometrial cancer cells improved the migrative and invasive abilities of endometrial cancer cells via the regulation of the miR-381-3p/E2F transcription factor 3 (E2F3) axis.^139^ Exosomes originating from the CAFs facilitated the invasive ability of endometrial cancer cells compared with the exosomes obtained from the normal fibroblasts (NFs), which was partially attributed to the low expression levels of miR-148b in exosomes of CAFs.^140^ Nevertheless, the proliferation of endometrial cancer cells and tube formation of endothelial cells were inhibited *in vitro* by the exosomal miR-499 derived from the mesenchymal stem cells, and the exosomal miR-499 also suppressed the tumor growth and angiogenesis *in vivo*.^141^ In summary, exosomes secreted by different cell types have diverse effects on endometrial cancer progression.

### Role of exosomes derived from body fluids in endometrial cancer

A total of 114 miRNAs were dysregulated in exosomes purified from the peritoneal lavage fluid of endometrial cancer patients in contrast to miRNAs in the exosomes isolated from the ascitic fluid of control patients, and the downregulated expression of peritoneal lavage exosomal miRNA-383-5p, miRNA-10b-5p, miRNA-34c-3p, miRNA-449b-5p, miRNA-34c-5p, miRNA-200b-3p, miRNA-2110, and miRNA-34b-3p potentially served as individual diagnostic biomarkers for endometrial cancer.^142^ Compared with the exosomes isolated from the urine of patients with symptoms of endometrial cancer, but no diagnosis of endometrial cancer, elevated miRNA-200c expression was discovered in exosomes isolated from the urine of endometrial cancer patients.^143^ Increased expression of 209 circRNAs and decreased expression of 66 circRNAs were discovered in exosomes isolated from the serum of stage III endometrial adenocarcinoma patients versus exosomes isolated from the serum of healthy individuals.^144^ Thus, nucleic acids carried by exosomes isolated from the peritoneal lavage fluid, urine, and serum of endometrial cancer patients have potential as novel diagnostic biomarkers of endometrial cancer.

Coculture of endometrial cancer cells and the exosomes isolated from the serum of polycystic ovary syndrome (PCOS) patients increased the migrative and invasive abilities of endometrial cancer cells, which may be regulated by the serum exosomal miR-27a-5p.^145^ Exosomal lectin galactoside-binding soluble 3-binding protein (LGALS3BP) was detected in exosomes isolated from the plasma of endometrial cancer patients and was upregulated with endometrial cancer progression, promoted the growth of endometrial cancer cells and facilitated the angiogenesis of HUVECs.^146^ The nucleic acids and proteins carried by exosomes may be involved in the progression of endometrial cancer.

## Conclusions and perspectives

**Figure 2:**
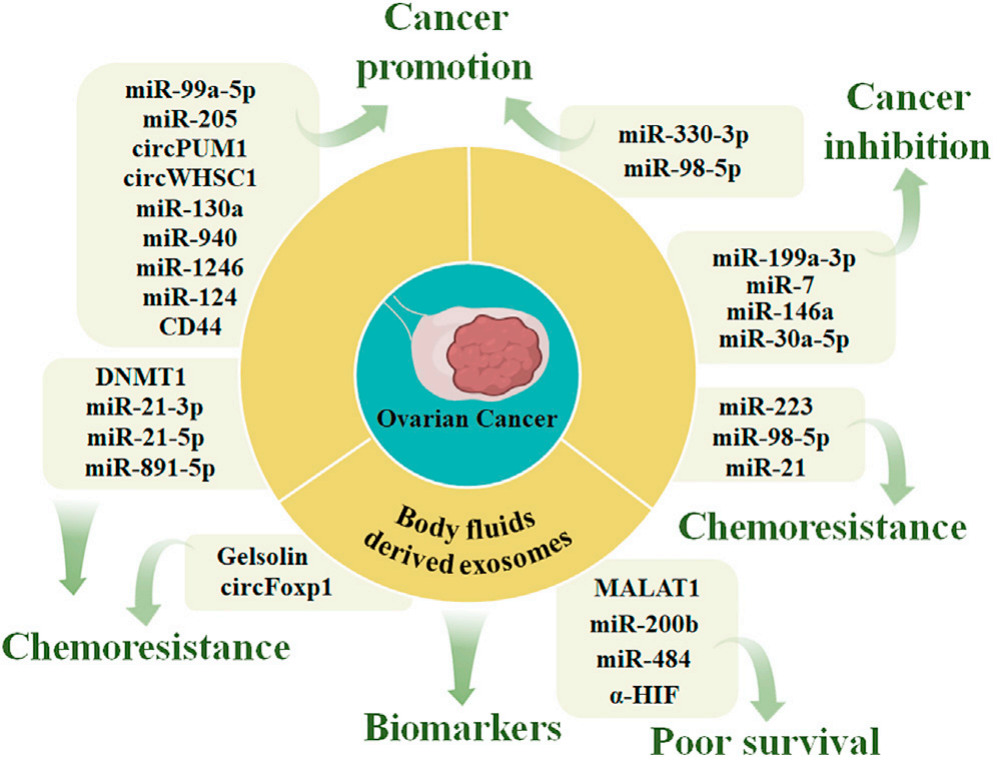
The role of exosomes in ovarian cancer

**Figure 3:**
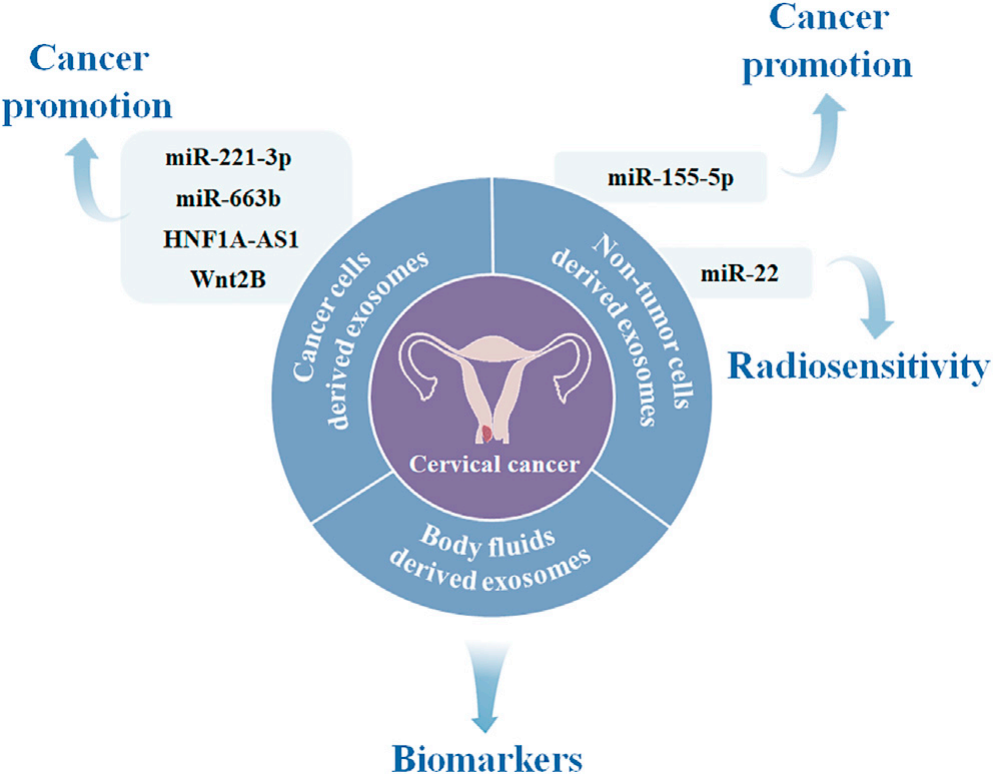
The role of exosomes in cervical cancer

**Figure 4:**
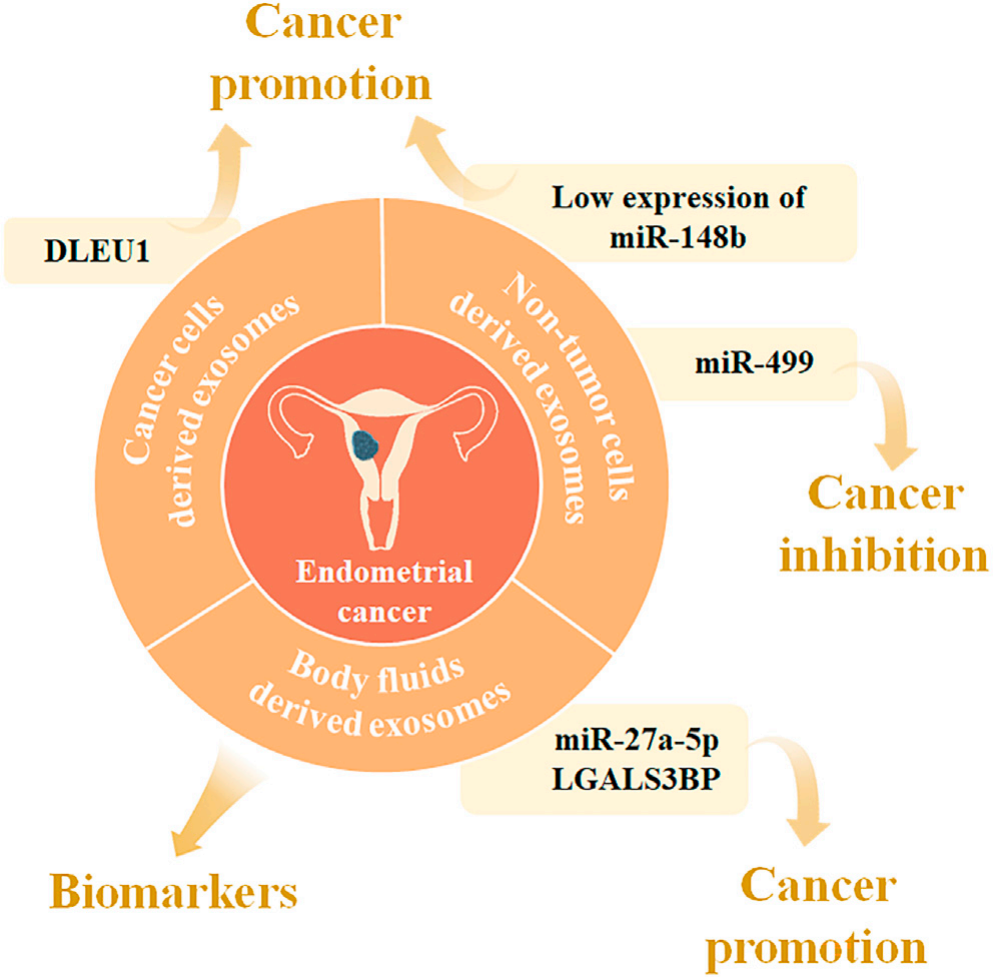
The role of exosomes in endometrial cancer

The small molecules within exosomes (including nucleic acids and proteins) allowed the identification of potential molecular biomarkers for the diagnosis, disease progression monitoring, and chemotherapy efficiency of ovarian cancer (Table 1; Figure 2), cervical cancer (Table 2; Figure 3), and endometrial cancer (Table 3; Figure 4).

**Table 1:** Exosome-carried nucleic acids and proteins as potential biomarkers for ovarian cancer

Type of biomarkers	Potential biomarkers	Exosome derivation	Reference
Nucleic acids	let-7 family, miR-200 family	cells	Miharu Kobayashi et al.^48^
	miR-99a-5p	cells	Yoshimura et al.^49^
	miR-205	cells	He et al.^50^; Wang et al.^51^
	circPUM1	cells	Guan et al.^53^
	circWHSC1	cells	Zong et al.^54^
	miR-940	cells	Chen et al.^57^
	miR-1246	cells	Kanlikilicer et al.^58^
	miR-124	cells	Zhang et al.^59^
	miR-199a-3p	cells	Kobayashi et al.^60^
	miR-7	cells	Hu et al.^61^
	miR-330-3p	cells	Yang et al.^62^
	miR-98-5p	cells	Guo et al.^63^
	miR-101	serum	Xu et al.^64^
	miR-146b-5p	serum	Wu et al.^65^
	miR-106a-5p, miR-122-5p, miR-185-5p	plasma	Zhang et al.^66^
	circ-0001068	serum	Wang et al.^67^
	miR-34a	serum	Maeda et al.^68^
	miR-214-3p	serum	Yang et al.^70^
	MtDNA	plasma	Keseru et al.^71^
	miR-1307, miR-375	serum	Su et al.^72^
	miR-145, miR-200c	serum	Kim et al.^73^
	miR-484	serum	Zhang et al.^74^
	miR-200b	plasma	Panet al.^75^
	lncRNA MALAT1	serum	Qiu et al.^76^
	miR-30a-5p	urine	Zhouet al.^77^
	miR-21	ascites	Cappellesso et al.^78^
	miR-6780b-5p	ascites	Cai et al.^79^
	miR-6126	cells	Kanlikilicer et al.^98^
	lncRNA UCA1	serum	Li et al.^99^
	circRNA Cdr1as	serum	Zhao et al.^100^
	circFoxp1	serum	Luo et al.^101^
	miR-891-5p	cells	Alharbi et al.^103^
	miR-223	cells	Zhu et al.^104^
	miR-21	cells	Au Yeung et al.^106^
	miR-146a	cells	Liya et al.^107^
	miR-30a-5p	cells	Liu et al.^108^
Proteins	FRα	plasma	Zhang et al.^86^
	HGF, STAT3, IL-6	serum	Dorayappan et al.^87^
	LBP, fibrinogen gamma chain, fibrinogen alpha chain	plasma	Zhang et al.^88^
	gelsolin	plasma	Zhang et al.^88^; Asare-Wereheneet al.^91^; Meshach Asare-Werehene et al.^105^
	MAGE 3/6, TGF-β1	plasma	Szajnik et al.^89^
	CD44	cells	Shen et al.^90^; Nakamura et al.^92^
	aHIF	serum	Tang et al.^93^
	glucose-6-phosphate dehydrogenase, transketolase, transaldolase	cells	Yi et al.^94^
	EGFR, HER2, CA125, FRα, CD24, EpCAM, CD9, CD63	plasma	Zhang et al.^96^
	COL5A2, LPL	cells	Cheng et al.^97^

**Table 2:** Exosome-carried nucleic acids and proteins as potential biomarkers for cervical cancer

Type of biomarkers	Potential biomarkers	Exosome derivation	Reference
Nucleic acids	miR-221-3p	cells	Zhang et al.^115^; Wu et al.^116^
	miR-663b	cells	You et al.^117^
	let-7d-5p, miR-20a-5p, miR-378a-3p, miR-423-3p, miR-7-5p, miR-92a-3p, miR-21-5p	cells	Honegger et al.^119^
	lncRNA HNF1A-AS1	cells	Luo et al.^120^
	miR-155-5p	cells	Li et al.^123^
	miR-22	cells	Konishi et al.^124^
	miR-146a-5p, miR-151a-3p, miR-2110	plasma	Ma et al.^125^
	miR-125a-5p	plasma	Lv et al.^126^
	let-7d-3p, miR-30d-5p	plasma	Zheng et al.^127^
	lncRNA DLX6-AS1	serum	Ding et al.^128^
	miR-146a, miR-21	cervicovaginal lavages	Liu et al.^130^
	lncRNAs HOTAIR, MALAT1, MEG3	cervicovaginal lavages	Zhang et al.^131^
Proteins	Patched1, Smoothened, Sonic hedgehog, Indian hedgehog	cells	Bhat et al.^121^
	Wnt2B	cells	Liang et al.^122^
	PI3k/Akt/mTOR	vaginal secretions	Zhang et al.^129^

**Table 3:** Exosome-carried nucleic acids as potential biomarkers for endometrial cancer

Type of biomarkers	Potential biomarkers	Exosome derivation	Reference
Nucleic acids	miR-133a	cells	Shi et al.^138^
	lncRNA DLEU1	cells	Jia et al.^139^
	miR-148b	cells	Li et al.^140^
	miR-499	cells	Jing et al.^141^
	miRNA-383-5p, miRNA-10b-5p, miRNA-34c-3p, miRNA-449b-5p, miRNA-34c-5p, miRNA-200b-3p, miRNA-2110, miRNA-34b-3p	peritoneal lavage	Roman-Canal et al.^142^
	miRNA-200c	urine	Srivastava et al.^143^
	miR-27a-5p	serum	Xiaoxia et al.^145^
Protein	LGALS3BP	plasma	Song et al.^146^

In contrast to other nanoparticles, exosomes present considerable advantages, including low immunogenicity, low toxicity, biological barrier permeability, high stability, excellent biocompatibility, and targetability, which render exosomes strong alternative candidates for clinical treatment.^147^ However, several challenges may be encountered during the clinical application of exosomes. First, the extraction and storage processes of exosomes must be optimized, which will benefit large-scale production of exosomes for clinical use. Second, the efficiency of drug loading and drug release of the exosome-based drug delivery system needs to be improved. Third, exosomes derived from various sources exhibit different functions in patients with different physiological or pathological statuses, which require further exploration. Therefore, further in-depth understanding of the effects of exosomes derived from different cell types or body fluids on gynecologic cancers can provide a rational theoretical basis for the targeted clinical treatment of ovarian cancer, cervical cancer, and endometrial cancer.
