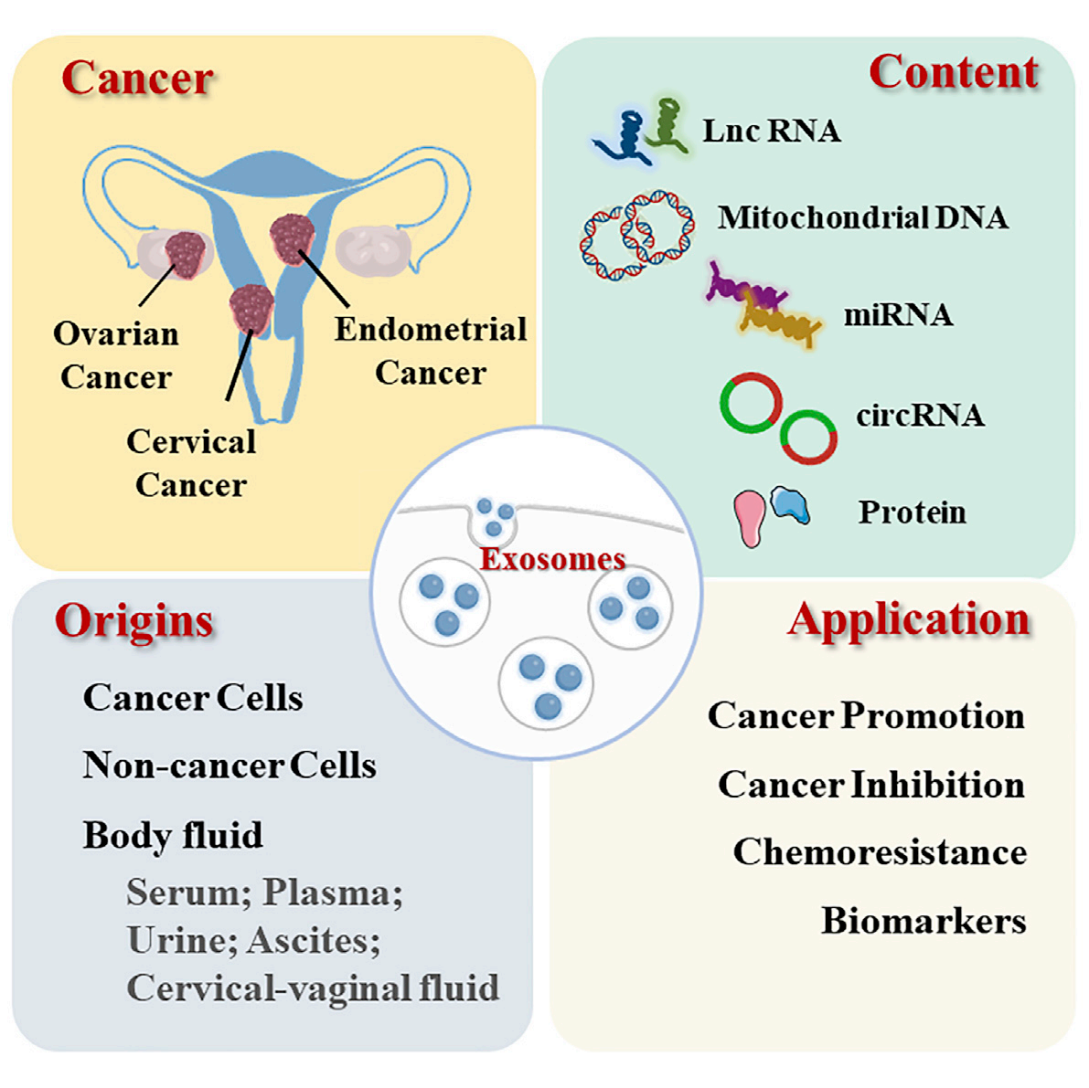# Nucleic acids and proteins carried by exosomes of different origins as potential biomarkers for gynecologic cancers

**DOI:** 10.1016/j.omto.2022.05.006

**Published:** 2022-05-23

**Authors:** Miaomiao Ye, Jing Wang, Shuya Pan, Lihong Zheng, Zhi-Wei Wang, Xueqiong Zhu

## Main text

(Molecular Therapy: Oncolytics *24*, 101–113; March 2022)

In the originally published version of this article, in the graphical abstract, the location of cervical cancer was misplaced in the vagina. This has now been corrected.

The authors regret this error.

**Figure:**